# Assessment of Anticancer Effects of *Aloe vera* on 3D Liver Tumor Spheroids in a Microfluidic Platform

**DOI:** 10.1002/bit.29033

**Published:** 2025-06-20

**Authors:** Atakan Tevlek, Gunes Kibar, Barbaros Cetin

**Affiliations:** ^1^ Department of Medical Biology Faculty of Medicine Atilim University Ankara Turkey; ^2^ Department of Materials Science and Engineering Micro Nano Particles (MNP) Research Group Adana Alparslan Turkes Science and Technology University Adana Turkey; ^3^ UNAM ‐ Institute of Materials Science and Nanotechnology Bilkent University Ankara Turkey; ^4^ Department of Mechanical Engineering Microfluidics & Lab‐on‐a‐chip Research Group Bilkent University Ankara Turkey

**Keywords:** 3D cell culture, *Aloe vera*, anticancer agent, microfluidic

## Abstract

The search for effective anticancer therapies has increasingly focused on natural compounds like *Aloe vera*, renowned for its therapeutic properties. This study investigates the anticancer properties of *Aloe vera* on 3D liver tumor spheroids via a PDMS‐based microfluidic device, providing a more physiologically realistic model compared to traditional 2D cultures. HepG2 cells were cultivated to generate 3D spheroids on‐chip, thereafter subjected to different concentrations of *Aloe vera* and the chemotherapeutic drug Doxorubicin to evaluate cytotoxic effects. The microfluidic system, validated by COMSOL simulations, facilitated continuous perfusion and real‐time assessment of cell viability over a duration of 10 days. The results indicated that *Aloe vera* markedly diminished cell viability by triggering apoptosis at concentrations over 12.5 mg/mL. IC50 values were determined at 72 h: 25 ± 0.10 mg/mL for *Aloe vera* and 5.47 ± 0.03 µg/mL for Doxorubicin in 2D cultures, but in 3D cultures, the IC50 values were 31.25 ± 0.14 mg/mL for *Aloe vera* and 8.33 ± 0.05 µg/mL for Doxorubicin. This study underscores the promise of *Aloe vera* as a natural anticancer agent and illustrates the efficacy of microfluidic platforms for enhanced drug screening and customized medicine applications.

## Graphical abstract

The comparative effects of Doxorubicin and *Aloe vera* on HepG2 spheroids grown in a microfluidic device are illustrated in the graphical abstract. In the upper panel, red markers indicate that Doxorubicin treatment primarily induces necrosis in the spheroids. In contrast, the bottom panel shows that low concentrations of *Aloe vera* (up to 12.5 mg/mL) enhance spheroid viability. At higher concentrations, however, *Aloe vera* begins to induce apoptotic and necrotic effects, reflecting a dose‐dependent shift in its biological activity. This biphasic response is consistent with the concept of hormesis, wherein low doses of a compound may exert stimulatory or adaptive effects, while higher doses become cytotoxic. In a microfluidic 3D culture setting, this study highlights the potential of *Aloe vera* to modulate cell survival and death pathways in liver cancer spheroids depending on the applied concentration.

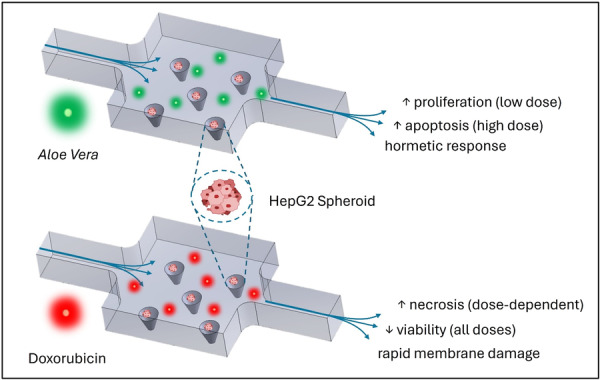

## 1 Introduction

Cancer is still a major global public health challenge as the second‐leading cause of death, with far‐reaching repercussions not only for individuals but also for their families and communities (Siegel et al. 2024). The urgent need to develop effective treatments for this complex and multifaceted disease has driven substantial research efforts to elucidate the underlying mechanisms of carcinogenesis, identify novel therapeutic targets, and develop innovative treatment strategies (Joshi et al. 2024). Progress in anticancer research is expected to improve survival rates and revolutionize early detection methods, providing more opportunities for intervention before cancer progresses to advanced stages. This progress also stems from uncovering key molecular pathways involved in cancer development and progression, all while fostering collaboration across multiple disciplines in the fight against cancer. Researchers are paving the way for the creation of more precise and personalized treatments. These efforts are supported by a growing emphasis on multidisciplinary collaboration, which is crucial for translating scientific discoveries into clinical applications (Carlberg and Velleuer 2021).

Anticancer agents are central to these efforts. These agents are specifically designed or selected for their ability to target and inhibit the growth of cancer cells while minimizing harm to normal cells (Naeem et al. 2022). Various anticancer agents are currently available, such as chemotherapeutic medicines (Yadav et al. 2021), targeted therapies (Min and Lee 2022), immunotherapies (Petroni et al. 2021), nanoparticles (Kibar et al. 2023), and natural compounds (Naeem et al. 2022). The inclusive goal of these agents is to disrupt various aspects of cancer growth and progression. Consequently, there is a continuous drive to discover new anticancer drugs, improve existing therapies, and explore novel combination strategies to enhance treatment efficacy and reduce side effects.

*Aloe vera*, a succulent plant with a long history of traditional medicinal use, has attracted attention for its anticancer potential in recent years. Several studies have investigated various bioactive compounds found in *Aloe vera*, including polysaccharides, anthraquinones, flavonoids, saponins, and enzymes, to assess their anticancer potential (Andrea et al. 2020; Palaniyappan et al. 2024). To date, experimental findings have revealed that *Aloe vera* extracts and isolated compounds exhibit anticancer activity against a range of cancer types, including breast, colon, liver, lung, and skin cancers, by causing apoptosis, modulating the immune system, reducing inflammation, inhibiting angiogenesis and the cell cycle, and DNA repair inhibition (Catalano et al. 2024).

In the last decade, *Aloe vera* has been explored in various forms—whole plant extracts, leaf extracts, gel, pulp, and powder—in cancer research (Kumar et al. 2019). For example, the lyophilized form of *Aloe vera* extract was assessed in HepG2 cells to demonstrate as both time‐ and dose‐dependent anticancer activity (Shalabi et al. 2015). This extract upregulated the P53 tumor suppressor gene and downregulated the Bcl‐2 antiapoptotic gene, indicating its potential to induce cancer cell death in both HepG2 and MCF7 cells by modulating these critical pathways (Bagherian et al. 2021; Shalabi et al. 2015). Similarly, another study investigating the anticancer properties of ethanolic leaf extracts of *Aloe vera* on the human liver (HepG2), cervical (HeLa), and lung (A549) cancer cell lines demonstrated potent proliferation‐inhibitory activity across all three cell lines, suggesting the need for further investigation into the subcomponents of *Aloe vera* (Algarni 2021). Later, a study testing *Aloe vera* plant extracts on human breast (MCF‐7) and lung (A‐549) cancer cell lines reported significant inhibition of cell proliferation, induction of apoptosis through reactive oxygen species (ROS) generation, cell cycle arrest, and DNA damage, highlighting *Aloe vera* as a potential natural source of anticancer compounds (Farshori et al. 2022). Laux et al. (2022) recently demonstrated that *Aloe vera* gel exhibits significant anti‐melanoma activity against A375 melanoma cells; however, its selectivity was reported to be lower than standard anticancer drugs like dacarbazine. Subsequent studies on isolated bioactive compounds from *Aloe vera*, such as Aloe‐emodin—an anthraquinone—have shown that it can enhance radiosensitivity in cancer cells by inducing apoptosis and inhibiting proliferation in HeLa cervical cancer and hepatocellular carcinoma cell lines (Zhu et al. 2023). In addition, Luo et al. (2024) reported that Aloe‐emodin can suppress breast cancer and non‐small lung cancer migration, invasion, and metastasis by triggering apoptosis. Chemical investigations revealed that *Aloe vera* contains a diverse array of polysaccharides and phenolic compounds, particularly anthraquinones. Notably, the International Agency for Research on Cancer (IARC) has classified *Aloe vera* whole leaf extract as a possible human carcinogen (Group 2B) (Guo and Mei 2016), indicating that its safety profile warrants cautious consideration. Despite promising preliminary results, further research is needed to fully establish the efficacy and safety of *Aloe vera* in cancer treatment (Wu et al. 2021).

To evaluate the efficacy and safety of anticancer compounds, it is crucial to develop advanced, physiologically relevant cell culture models that bridge the gap between in vitro experiments and clinical outcomes (Tosca et al. 2023). In recent years, advancements in three‐dimensional (3D) cell culture systems and microfluidic technologies have significantly enhanced cancer research by enabling the creation of more accurate models that mimic the complex tumor microenvironment (Law et al. 2021). 3D systems allow for better cell‐cell and cell‐extracellular matrix interactions compared to traditional two‐dimensional (2D) culture models (Kapałczyńska et al. 2018), while microfluidic devices provide precise control over the cellular microenvironment and enable high‐throughput drug screening (Cardoso et al. 2023). These platforms also support miniaturization, coculture setups, real‐time imaging, and point‐of‐care diagnostics, all contributing to the development of more personalized and effective therapies (Tevlek et al. 2023). In particular, microfluidic 3D cell culture systems have been widely employed to assess the toxicity of drugs, particularly by modeling liver‐like tissue environments (Khot et al. 2020; Moradi et al. 2020; Polidoro et al. 2021). In the study by Do et al. (2023), a microfluidic device made from polymethylmethacrylate (PMMA) was developed to monitor the growth of 3D HepG2 cell cultures and evaluate drug delivery systems. In the work by Taroncher et al the microfluidic device was fabricated using a silicon wafer as a master mold. This approach necessitated specialized tools and controlled conditions, including cleanroom facilities, spin‐coating, and baking steps (Taroncher et al. 2024). In a recently reported study by Hoyos‐Vega et al. (2024), a microfluidic device with an integrated droplet‐based bioanalysis unit was developed to analyze picoliter volumes of cell‐conditioned media allowing noninvasive glucose and albumin detection in hepatic 3D cultures. The device featured 140 microwells for spheroid formation, along with a droplet generator and micromechanical valves for detecting glucose and albumin via enzymatic and immunoassays.

The aim of this study is to design and develop a cost‐effective, reproducible, and biocompatible microfluidic device for 3D cell culture, which can be utilized to study the effects of anticancer agents (such as *Aloe vera*) on tumor spheroids. To accomplish this, we introduce a novel approach for generating HepG2 spheroids using a microfluidic device to evaluate, for the first time, the anticancer efficacy of *Aloe vera* compared to the well‐known chemotherapeutic agent, Doxorubicin. Polydimethylsiloxane (PDMS) was selected as the material for the device due to its advantageous properties, including biocompatibility, optical transparency, and gas permeability, all of which make it highly suitable for cell culture applications (Khot et al. 2020). Furthermore, PDMS's inherent hydrophobic nature and surface characteristics facilitate the formation of 3D cell structures without the need for scaffolds. Significant efforts have also been made to design lab‐on‐a‐chip liver models using PDMS‐based microfluidic devices, enabling more effective toxicity analysis (Polidoro et al. 2021). The mold of the microfluidic device was fabricated through mechanical machining, which is a practical and economical alternative to lithography‐based techniques that often require clean‐room equipment, costly and time‐consuming (Çetin et al. 2014; Zeinali et al. 2015). Moreover, once the mold is fabricated out of metal or polymer, the mold may be used repeatedly in the fabrication. A comprehensive series of experiments—including COMSOL simulation analysis and multiple cell viability and cytotoxicity assays such as live‐dead imaging, glucose consumption measurement, lactate dehydrogenase (LDH) activity, and albumin secretion analysis—demonstrates the feasibility and clear advantages of this approach for 3D spheroid formation within a microfluidic flow system. In addition, the performance of our 3D cell culture is also compared to that of 2D cell culture methods.

## 2 Materials and Methods

### 2.1 Design and Simulation

**Figure 1:**
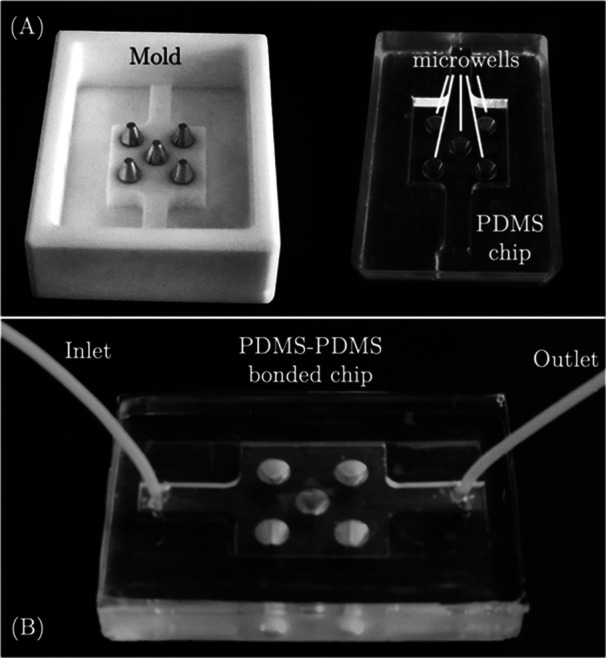
(A) Teflon master mold and the fabricated PDMS chip, and (B) the sealed chip with PTFE tubings. PDMS, polydimethylsiloxane.

**Figure 2:**
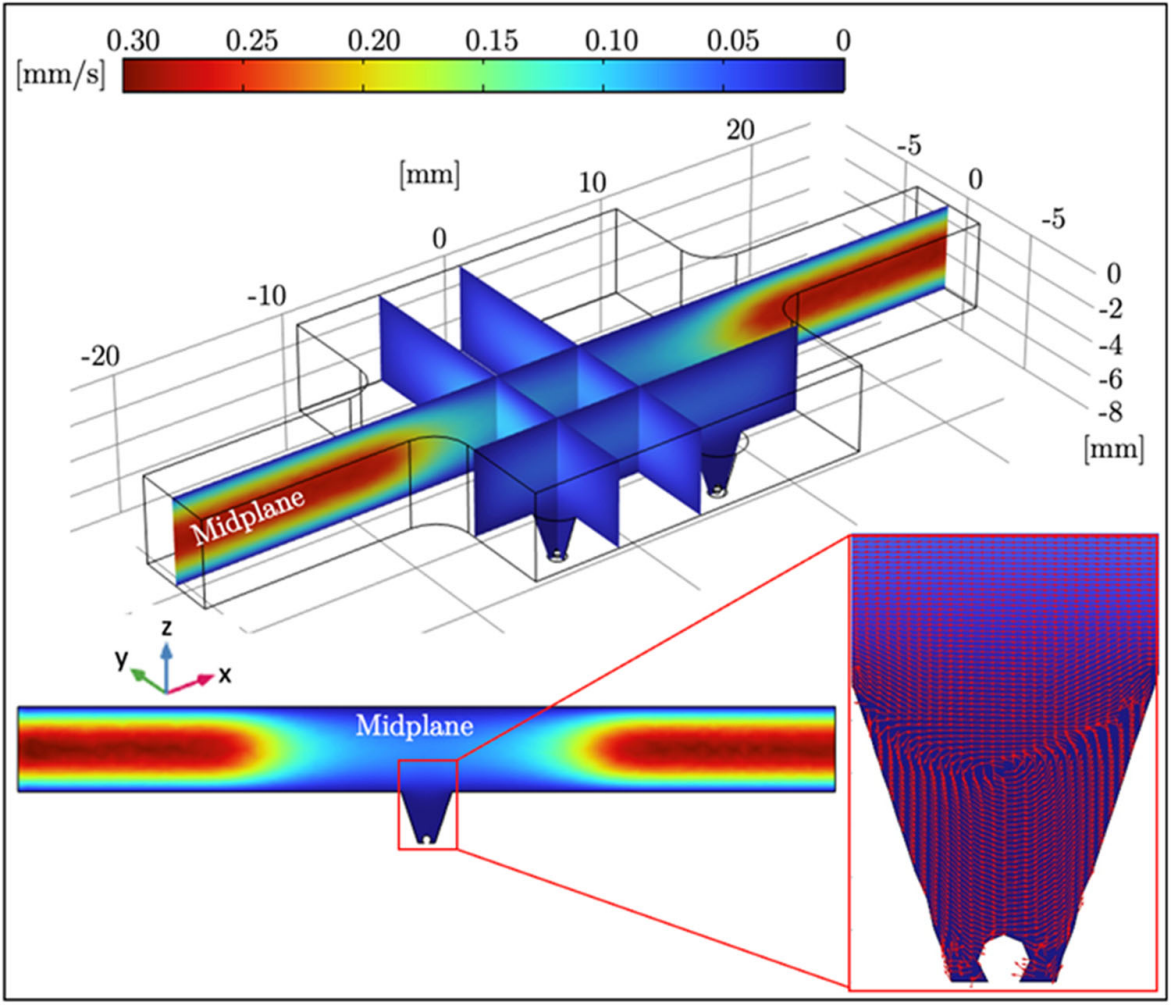
COMSOL multiphysics simulation showing the velocity profile and flow field distribution inside the microfluidic device. The color scale indicates fluid velocity from 0 mm/s (blue) to 0.30 mm/s (red). The flow enters from the left inlet and exits through the right outlet, maintaining a smooth laminar profile along the straight channel. The magnified view of the truncated cone section at the midplane highlights the controlled decrease in velocity, minimizing shear stress in the 3D cell culture wells.

A single microfluidic device containing a straight flow channel connecting the inlet and outlet and was developed in this study. SolidWorks (Dassault Systèmes, Vélizy‐Villacoublay, France) was used to create the CAD model of the microfluidic device. 3D cell culturing wells are placed in the mid‐section of the device (please see Figure 1). The width of the straight flow channel entering and exiting the reservoir unit located in the mid‐section was selected as 5 mm. The Navier‐Stokes equations were utilized to assess the flow field in the microfluidic device through a COMSOL Multiphysics (version 5.2) model. No‐slip boundary conditions were assigned on the channel wall, volumetric flow rate was defined at the inlet, and zero pressure boundary conditions were assigned on the outlet of the microchannel. Using different mesh settings, mesh independency of the simulations were asured. Following several simulations, the height of the truncated cone was determined as 5 mm, while the radius of the top and bottom circular base was 0.5 mm and 2 mm, respectively (which corresponds to a final cone volume of 27.49 µL). The flow field inside the final design of the microfluidic device is illustrated in Figure 2.

### 2.2 Fabrication of Microfluidic Device

The fabrication of the microfluidic device was performed by following PDMS‐modling (Zeinali et al. 2015). A reusable PTFE master mold (50 mm × 70 mm) containing five well‐forming truncated cones was designed and produced by mechanical machnining (Universal Milling Machine‐TOS, Czech Republic). Commercially available Sylgard 184 elastomer kit (Dow Corning, USA) was used according to the manufacturer's instructions by mixing 10 parts base to 1 part curing agent (w/w). All samples were mixed thoroughly with a magnetic bead under a magnetic stirrer (ISOLAB GmbH, Germany) at a speed of 200 rpm for 1 min at room temperature. Subsequently, the resulting uncured mixture was poured into the Teflon mold and plastic petri dishes (120 × 17 mm, ISOLAB GmbH, Germany) separately and was degassed by a desiccator to remove the air bubbles for 45 min at room temperature. Lastly, the samples were cured at 80°C for 80 min in a vacuum oven (Nuve EV018, Turkey). At the end of the curing time, the cured samples were peeled by using a spatula when they cooled at room temperature. Subsequently, the PDMS membranes were subjected to purging with pressurized nitrogen gas within a fume hood, followed by exposure to oxygen plasma treatment (Atto, Diener, Germany) for 90 s under the working condition of 100 W, 50 mL/min oxygen flow, and 0.3 mBar to increase their surface energy with objective the objective of promoting irreversible bonding between the two membranes. Lastly, the PDMS membranes were sealed with their treated surfaces facing each other and fixed conformal contact by gently crushing between thumb and index fingers for a duration of 10–15 s at room temperature. Before the sealing process, the inlet and outlet sections were opened on the upper PDMS membrane with the help of a 1.25 mm PDMS puncher (Elveflow, France). Then, the PTFE tubing (1/16'' OD × 1/32´´ ID) (Elveflow, France) was inserted into the inlet and outlet. Figure 2 shows the developed master mold, the PDMS chip, and the sealed chip with tubing.

### 2.3 Cell Maintenance

Human hepatocellular carcinoma cell line (HepG2) (HB8065, ATCC, UK) was used in this study. The cells were cultivated in standard cell culture dishes under controlled conditions of 37°C and 5% CO_2_. The culture media used was Dulbecco's modified Eagle's medium (DMEM) High Glucose (D6429, Sigma Aldrich, Germany) supplemented with 10% fetal bovine serum (FBS) (FBS‐12A, Capricorn, Germany), 1% antibiotic‐antimycotic solution (A5955, Sigma Aldrich, Germany). Once the cells had reached confluence, they were rinsed with Dulbecco's phosphate‐buffered saline (DPBS) (Biological Industries, USA) and subsequently passaged. The cell passage was accomplished using a trypsin solution (Trypsin EDTA Solution A (0.25%), EDTA (0.02%) (Biological Industries, USA), which was afterward neutralized with the prepared culture medium mentioned above. Subsequently, the cell suspension solution underwent centrifugation at 2500 rpm for 2. 5 min, following which the cells were re‐suspended in the culture medium. The concentration of the cell suspensions was determined by using an automated cell counter (EVE Cell Counter, NanoEntek, Korea).

### 2.4 3D Spheroid Formation and Culture

Tumor spheroid formation in the microfluidic device was demonstrated by HepG2 cells. Before conducting the experiment, the microfluidic device and PDMS membranes underwent a series of cleaning and sterilization procedures. First, they were washed with deionized water, 70% ethanol, and phosphate‐buffered saline (PBS) for 5 min each and dried using autoclaved paper filters. Subsequently, both sides of the devices were subjected to UV treatment for 30 min to ensure sterilization. Sterile samples were gently placed in the oxygen plasma device for surface modification without compromising their sterilization in the petri dish, and at the end of the process, they were taken back into the laminar cabinet for cell cultivation in a sterile manner. Following the sterilization procedure, a 0.5% (w/v) polyvinyl alcohol (PVA) (Sigma Aldrich, USA) solution in deionized water was utilized to coat the entirety of the inner surface of the PDMS chip. This coating was left undisturbed for 30 min to prevent cell adherence to the chip's surface. At the end of the incubation period, the solution was fully scraped from the chip surface, and 10^3^ HepG2 cells were seeded into each conical well of the chip in a 30 µL of culture medium. The treated surfaces were afterward promptly sealed without any delay, and the cell‐seeded chip was incubated for 6 h without any disturbance to allow the initial cellular cross‐talk and organization. At the end of the incubation period, the tubing was inserted into the chip under sterile conditions, and the culture medium was fed into the microfluidic device at 200 µL/h using a syringe pump (Harvard Apparatus, PHD Ultra, USA). In parallel, spheroid formation in the microfluidic device was compared with a well‐known conventional method, hanging drop. Droplets of the same cell number and volume were carefully placed on the lid of a 60 mm plastic petri dish (ThermoFisher Scientific, USA). To prevent the droplets from drying, 6 mL of d‐PBS (ThermoFisher Scientific, USA) was added to the bottom of the petri dishes. Subsequently, both the cell suspensions prepared using the hanging drop technique and those formed within the chip were placed in a CO_2_ incubator (Memmert ICO105, USA) set at 37°C and cultured for 7 days. The selected cell number, incubation duration before flow and the flow rate in the microfluidic chip were adopted based on previous microfluidic studies that reported optimal spheroid formation and stable cell viability using comparable settings (Miyamoto et al. 2015; Zuchowska et al. 2017).

### 2.5 Biological Studies

**Figure 3:**
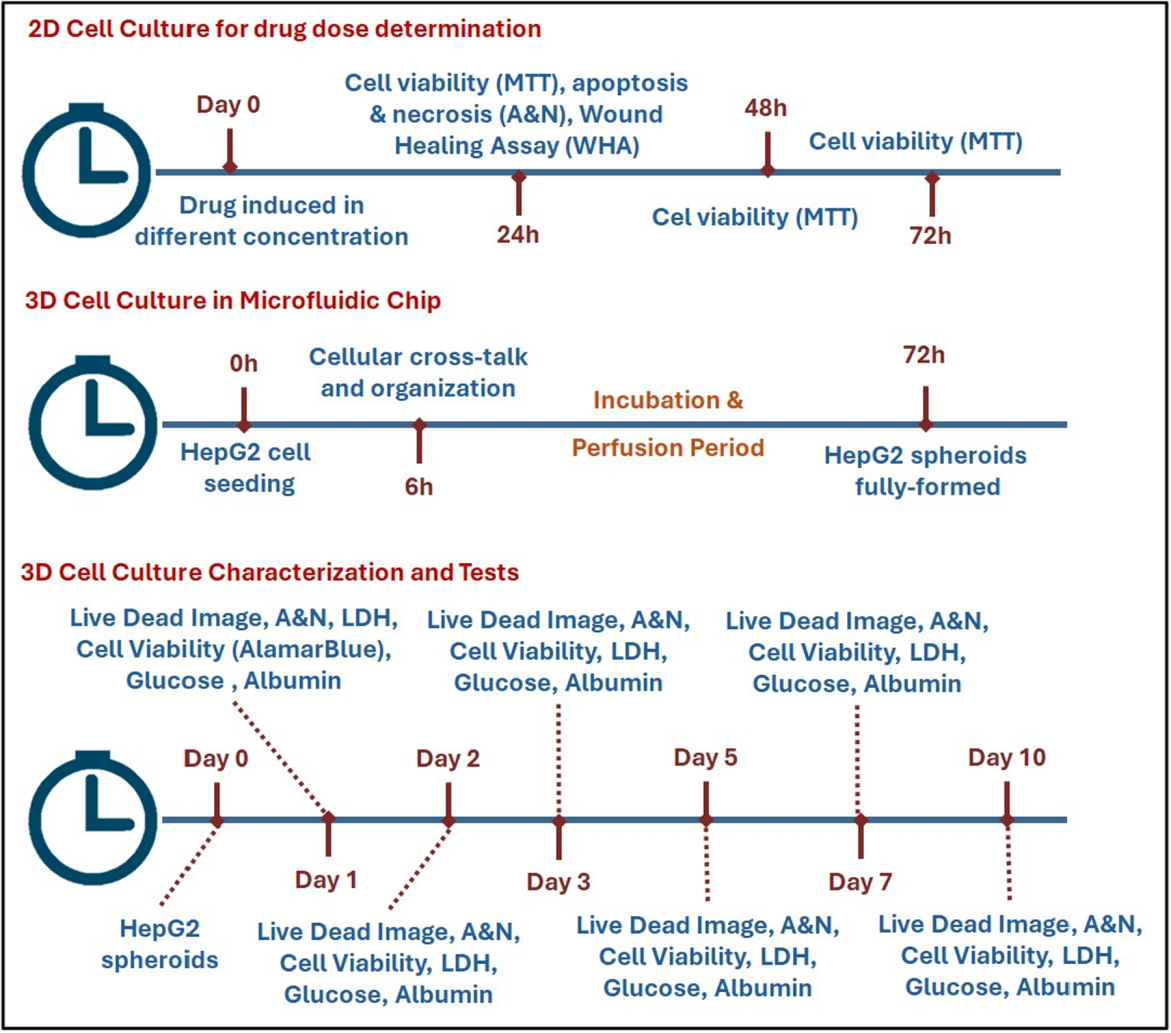
Schematic representation of the experimental design for two‐dimensional (2D) and three‐dimensional (3D) cell culture studies. LDH, lactate dehydrogenase; MTT, 3‐(4,5‐dimethyl‐2‐thiazolyl)‐2,5‐diphenyl‐2H‐tetrazolium bromide.

To provide a comprehensive overview of the experimental design and the progression of biological tests performed throughout the study, a schematic representation has been developed. This figure outlines the timeline and experimental setup for both 2D and 3D cell culture studies, including the drug dose determination, cytotoxicity analysis, and characterization assays conducted on HepG2 spheroids. These assays encompass key measurements such as cell viability, apoptosis/necrosis, LDH activity, glucose consumption, and albumin secretion across multiple time points. The schematic, presented in Figure 3, offers an accessible visual summary of the methodology used to evaluate the effects of Doxorubicin and *Aloe vera* on HepG2 cells in both 2D and 3D culture environments.

#### 2.5.1 Cytotoxicity Analysis

Doxorubicin (Doxorubicine Hydrochloride, Sandoz, Turkey) and *Aloe vera* (Terra‐Pure™ Freeze Dried *Aloe vera* Juice Powder 200X, TN003, Melbourne, ABD) solutions were prepared in sterile PBS. To figure out the IC50 value of the Doxorubicin and *Aloe vera* solution on HepG2 cells, a volume of 100 μL containing HepG2 cells at a density of 1 × 10^3^ was introduced into each well of a 96‐well plate for 2D cytotoxicity analysis. Subsequently, the plate was incubated at 37°C for 24 h in 5% CO_2_% and 95% humidity in an incubator (Memmert ICO105, USA). The cells were subjected to various concentrations of Doxorubicin (0, 0.75, 1.5, 3.125, 6.25, 12.5, and 25 μg/mL) and *Aloe vera* (0, 3.125, 6.25, 12.5, 25, 50, and 100 mg/mL). The drug underwent dilution using a culture medium, and each treatment was replicated in five microwells, with a volume of 200 μL per well. The samples were then incubated for 24, 48, and 72 h. Cell viability was assessed by employing a 3‐(4,5‐dimethyl‐2‐thiazolyl)‐2,5‐diphenyl‐2H‐tetrazolium bromide (MTT) reagent (ThermoFisher Scientific, United States). Briefly, 10 μL of MTT was introduced to the cells, followed by an additional incubation period of 4 h. The liquid was disposed of, adding 150 μL of dimethyl sulfoxide (DMSO) to each well. The absorbance of the wells was measured using a microplate reader (Multiskan Sky‐High Microplate Spectrophotometer, ThermoFisher Scientific, USA) at a wavelength of 570 nm, and the survival rate of cells was determined by comparing it to the negative control. Toxicity assessments were also performed using 3D models, akin to the 2D investigations. A solution consisting of 10% MTT reagent was introduced into the chip, after which the flow was halted to enable interaction with the spheroids for 4 h. Then, the culture medium was removed with the help of the pumping system, DMSO was added to the spheroids in the chip, and 200 µL samples taken from the outlet section were read under similar conditions.

#### 2.5.2 Determination of Apoptosis and Necrosis

Acridine orange (AO)/propidium iodide (PI) staining was used to examine the apoptotic and necrotic effect of the treated molecules on HepG2 cells in 2D culture. The test was conducted on the third day of the culture for 2D culture studies and on the 1st, 3rd, 5th, 7th, and 10th days of culture for 3D culture studies. Briefly, 10^4^ cells were planted in each well of a 48‐well plate in the growth medium for 2D culture tests. After 24 h, the growth media was replaced with a fresh medium containing the same concentration of test compounds as in the cytotoxicity analysis. The culture medium was removed at the end of the incubation durations, and the cells were washed once with PBS (Sigma Aldrich, Germany). The cells were then stained with 200 L of a staining solution containing 25 µg/mL AO and 25 µg/mL PI for 30 s before being viewed using a fluorescence microscope with the FITC and Texas Red filters (Leica DM IL Led, Netherlands). On the analysis days mentioned above for 3D culture studies, the test chip was placed directly under the fluorescent microscope, and images of the spheroids were obtained as soon as the culture medium was withdrawn and the staining solution was fed. In 3D culture analyses, the evaluation of apoptosis and necrosis were evaluated by staining color and intensity of the cells, while in 2D culture analyses, the amount of apoptotic and necrotic cells were counted and calculated with the following equations:

(2) Apoptoticcells(%)=(Totalnumberofapoptoticcells/Totalcountedcells)×100,

(3) Necroticcells(%)=(Totalnumberofnecroticcells/Totalcountedcells)×100.

#### 2.5.3 Wound Healing Assay

The aim of the wound healing assay was to evaluate the effects of different doses of Doxorubicin and *Aloe vera* on the inhibition of cell motility. To achieve the desired objective, 2.5 × 10^4^ HepG2 cells were introduced into 24‐well plates. Following a 24‐h incubation period, the formed cell monolayer was subjected to a controlled scratch using a pipette tip. Subsequently, any residual cell debris was eliminated using a series of three washes utilizing PBS. Subsequently, the identical doses examined in the cytotoxicity assay were introduced into the wells and co‐incubated for an additional duration of 24 h. Subsequently, the scratches were captured using an inverted microscope, and the degree of closure throughout the widths was assessed to determine the pace of cell migration.

#### 2.5.4 Cell Viability Analysis in HepG2 Spheroids

The cell viability was determined using the alamarBlue Cell Viability Reagent (Invitrogen, ThermoFisher Scientific, USA) following the manufacturer's instructions. In brief, a culture medium containing 10% AlamarBlue solution was fed into the PDMS‐based chip and incubated with spheroids for 2 h by halting the flow. The supernatant was then collected from the outlet by continuing the flow, and 200 µL of samples were exemplified (*n* = 3, 5 parallel) and were measured at 560/590 nm with a microplate reader (Multiskan Sky‐High Microplate Spectrophotometer, ThermoFisher Scientific, USA). Cell viability inhibition as a percentage is calculated with the following equation:

(4) Cellinhibitionpercentage=[(viabilityofuntreatedsample−viabilityoftreatedsample)/viabilityofuntreatedsample]×100%.

#### 2.5.5 Lactate Dehydrogenase (LDH) Activity in HepG2 Spheroids

Spent cell culture media supernatant was collected from the microfluidic device on the first, third, fifth, seventh, and tenth days of culture until the end of the continuous flow of Doxorubicin and *Aloe vera* treatment period and stored at −20°C. The cell damage was evaluated by the LDH quantity released into the culture medium by the colorimetric lactate dehydrogenase (LDH) activity test (Elabscience, USA). Briefly, 250 µL of the collected supernatant was taken on the time points mentioned above, placed in a 6‐well plate, and exposed with the equivalent volume of the substrate buffer in the test kit. Subsequently, Coenzyme I solution in the test kit was added to all wells, and the culture dishes were incubated at 37°C for 15 min. Afterward, 250 µL of 2, 4‐dinitrophenylhydrazine solution was added to all wells and incubated for 15 min at 37°C. Then, 2.5 mL of 0.4 mol/L NaOH solution was added to all wells and incubated for 5 min at RT. In the end, 200 µL of samples were taken into a 96‐well culture dish as six parallel from each sample (*n* = 5) and read at 440 nm with a microplate reader (Multiskan Sky‐High Microplate Spectrophotometer, ThermoFisher Scientific, USA).

**Glucose Consumption in HepG2 Spheroids.** The glucose consumption test was also measured on the supernatant, similar to the lactate dehydrogenase determination, and samples prepared for lactate dehydrogenase were also used for this purpose. Glucose consumption in the spheroids was determined by a conventional glucometer (ACON Biotech, China). Briefly, 10 μL of culture medium was exemplified from the collected samples on the above‐mentioned time points and was directly read in the glucometer. The glucose consumption was calculated by the concentration difference between the fresh and spent cultures medium (*n* = 5).

**Albumin Secretion in HepG2 Spheroids.** The concentrations of albumin in the spheroid supernatants were measured by using a commercially available Colorimetric Albumin Assay Kit (Bromocresol Green Method) (Elabscience, USA) on the days of culture specified. The experiment was carried out following the manufacturer's instructions. A 15 µL sample of the collected supernatant was taken, and the albumin released by the spheroids was measured (*n* = 3, 5 parallel). To determine the quantitative albumin from the obtained absorbance values, a standard curve was established using the albumin solution contained in the kit, and the results were graphed.

### 2.6 Statistical Analysis

All statistical analyses were conducted to evaluate the effects of different treatment groups and time points on cell viability, LDH activity, glucose consumption, albumin secretion. Initially, a repeated‐measures analysis of variance (ANOVA) (two‐way) was performed to compare these biochemical activities across multiple time points (Day 1, Day 3, Day 5, Day 7, and Day 10) within each treatment group (Doxorubicin 5 µg/mL, Doxorubicin 10 µg/mL, *Aloe vera* 50 mg/mL, and *Aloe vera* 100 mg/mL). This analysis was chosen to account for the correlated nature of the repeated measurements taken from the same experimental units over time. It allowed us to determine whether there were significant differences in each biochemical activity over time within the groups. Additionally, a one‐way ANOVA was applied at each individual time point to determine statistically significant differences between treatment groups on a per‐day basis. Following the two‐way ANOVA, Tukey's multiple comparison test was used as a post‐hoc analysis to identify specific time points where significant differences between treatment groups occurred. Tukey's test was selected because it controls the familywise error rate and is appropriate when making multiple comparisons. All statistical analyses were performed using GraphPad Prism 6 software, and significance was set at *p* < 0.05. Results are presented as mean ± standard deviation unless otherwise stated. Different *p* values and their level of significance are as follows: *p < 0.05, **p < 0.01, ***p < 0.001, ****p < 0.0001. The data sets used and/or analyzed during the current study are available from the corresponding author on reasonable request.

## 3 Results and Discussion

COMSOL Multiphysics simulations provided valuable insights into the fluid dynamics within the microfluidic device, which play a critical role in maintaining appropriate conditions for the 3D cell cultures. The flow field, as shown in Figure 2, demonstrates a laminar and uniform flow profile along the straight channel, with peak velocities near the inlet and outlet regions. The velocity decreases smoothly toward the midsection where the 3D cell culture wells are located, minimizing shear stress on the cells. This controlled flow environment is crucial for ensuring stable cell growth and preventing any negative effects caused by excessive shear forces. The truncated cone design in the midsection further optimizes the flow, reducing the likelihood of dead zones and ensuring that nutrients and oxygen are evenly distributed to the cells. The simulation results confirm that the selected design parameters—such as the 5 mm width of the channel and the truncated cone's dimensions—create a well‐balanced flow field, essential for consistent experimental outcomes. The uniform flow conditions provide a stable foundation for the subsequent experiments evaluating the cytotoxic effects of *Aloe vera* and Doxorubicin on HepG2 cells.

**Figure 4:**
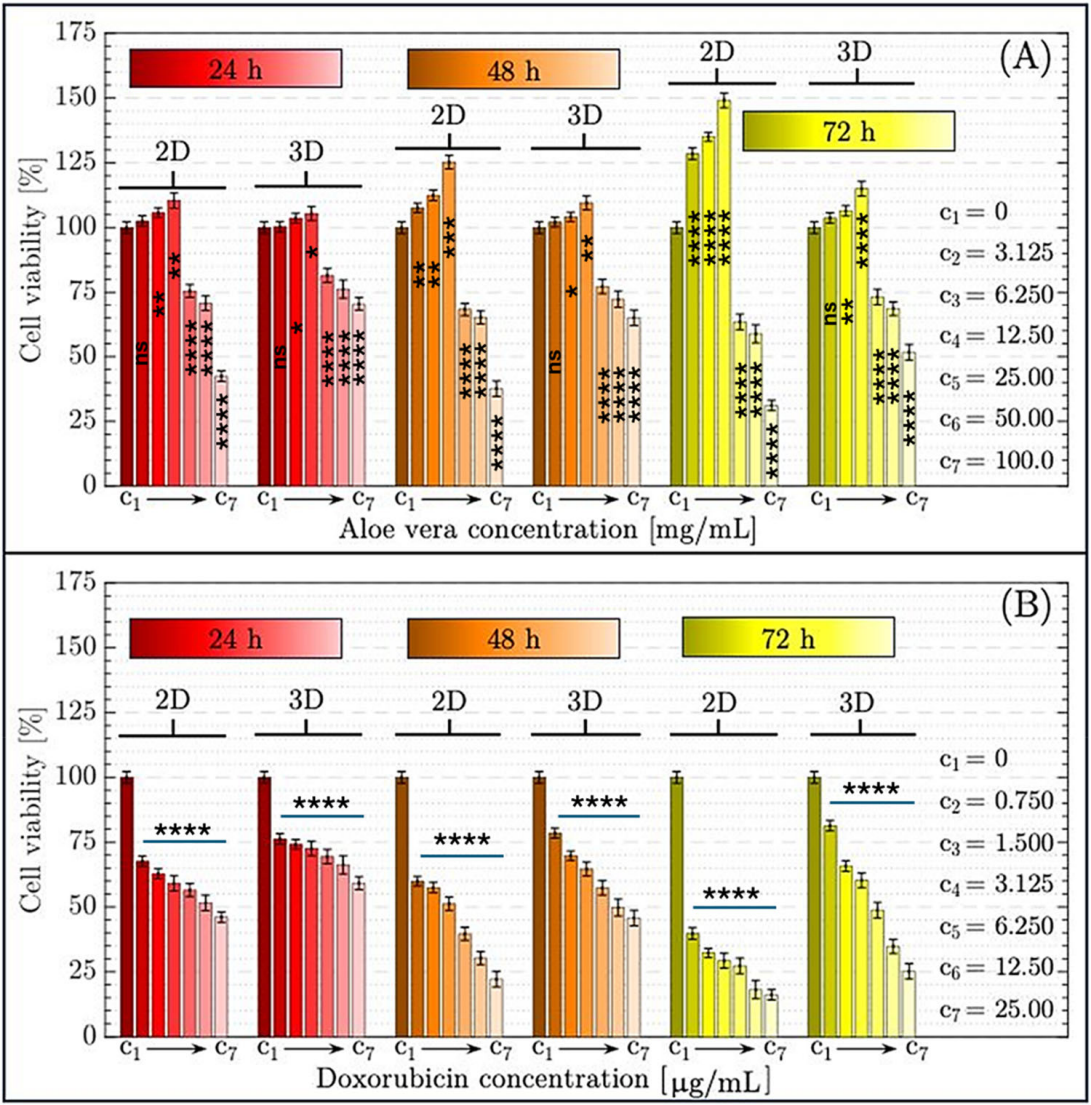
Cytotoxicity of *Aloe vera* (A) and Doxorubicin (B) on HepG2 cells in 2D and 3D cultures over 24, 48, and 72 h. Bar graphs represent cell viability percentages across a range of concentrations to illustrate dose‐ and time‐dependent responses. Each bar indicates the mean ± standard deviation of five replicates (*n* = 5). Statistical significance within each culture model (2D or 3D) was assessed using repeated‐measures two‐way ANOVA followed by Tukey's multiple comparisons test. Comparisons were made between each treatment group and the corresponding control (C1) at each time point. Asterisks above individual bars denote significant differences versus the control group (C1): *p < 0.05, **p < 0.01, ***p < 0.001, ****p < 0.0001; ns = not significant. 2D vs. 3D comparisons at the same concentration and time point were performed separately and are reported in Section 3 to avoid visual overcrowding in the figure.

The cytotoxic effects of *Aloe vera* and Doxorubicin on HepG2 cells were systematically analyzed to determine their respective IC50 values. Utilizing a two‐tier approach, cells were exposed to varying concentrations of each agent in both 2D and 3D culture models, as described in Section 2. The resulting data, depicted in Figure 4, illustrate the dose‐dependent viability of HepG2 cells over 24, 48, and 72‐h intervals. The calculated IC50 values are additionally presented in Table 1, providing a detailed quantitative assessment of the cytotoxic profiles for both compounds under different experimental conditions.

**Table 1:** The comparative IC50 values of *Aloe vera* and Doxorubicin on HepG2 cells in 2D and 3D culture systems.

	*Aloe vera* (mg/mL)			Doxorubicin (µg/mL)		
	24 h	48 h	72 h	24 h	48 h	72 h
2D	41.67 ± 0.15	33.33 ± 0.12	25.00 ± 0.10	8.33 ± 0.05	6.25 ± 0.04	5.47 ± 0.03
3D	58.33 ± 0.20	50.00 ± 0.18	31.25 ± 0.14	12.50 ± 0.07	10.42 ± 0.06	8.33 ± 0.05

The impact of *Aloe vera* on cell viability exhibited significant differences depending on both the concentration and the incubation time, as assessed in 2D and 3D cell culture models. At 24 h, C_4_ (12.5 mg/mL) produced the highest cell viability, reaching approximately 110% in 2D (*p* < 0.01) and approximately 105% in 3D cultures when compared with the control group (C_1_) (*p* < 0.05). At concentrations above C_4_ (> 12.5 mg/mL), viability progressively declined, dropping to around 25% at C_7_ (100 mg/mL) (*p* < 0.0001). At 48 h, the same C_4_ concentration resulted in further increased viability (~125% in 2D [*p* < 0.001], and ~115% in 3D [*p* < 0.01]), while higher doses again led to reduced viability (*p* < 0.001 to *p* < 0.0001 depending on dose). By 72 h, *Aloe vera* treatment at C_4_ peaked at approximately 150% in 2D (*p* < 0.0001) and approximately 125% in 3D (*p* < 0.001), marking the maximal viability across all time points. At concentrations beyond C_4_ (> 12.5 mg/mL), however, apoptotic and necrotic responses became evident, with viability decreasing significantly—reaching near 25% at C_7_ (*p* < 0.0001). This dose‐ and time‐dependent behavior aligns with the principle of hormesis, a biphasic dose–response phenomenon in which low doses of a compound can stimulate cell survival and proliferation, whereas higher doses become inhibitory or cytotoxic (Calabrese et al. 2025; Fonseca et al. 2025; Wang et al. 2024). These findings, on the other hand, align with a previous report which similarly demonstrated that *Aloe vera* promotes cellular viability at low concentrations but induces cytotoxic effects as the dose increases (Catalano et al. 2024).

The cytotoxic effects of Doxorubicin on HepG2 cell viability exhibited a clear dose‐ and time‐dependent pattern in both 2D and 3D cultures. Within the first 24 h, a substantial reduction in viability was observed beginning from C_2_ (*p* < 0.0001), with viability dropping to below 50% by C_3_, and further declining to approximately 25% at higher concentrations (C_6_–C_7_, *p* < 0.0001). At 48 h, the cytotoxic effect intensified: cell viability fell to nearly 25% even at intermediate concentrations (C_3_–C_4_), and approached zero at higher concentrations (C_6_–C_7_, *p* < 0.0001) in both 2D and 3D models. By 72 h, Doxorubicin‐induced cell death became even more pronounced. In 2D cultures, viability was nearly undetectable from C_5_ and above (*p* < 0.0001), while in 3D cultures, minimal residual viability (~10%–15%) persisted at some concentrations (C_6_–C_7_, *p* < 0.0001), indicating a slightly attenuated but still highly effective cytotoxic response in the 3D model. These findings confirm that Doxorubicin exerts strong and sustained cytotoxicity regardless of the culture system, although 3D spheroids may exhibit a modest degree of temporal resistance. Overall, the data are consistent with Doxorubicin's established profile as a potent chemotherapeutic agent capable of inducing rapid and extensive tumor cell death across a broad concentration range (Sun et al. 2025; Tabbasam et al. 2025).

In addition to within‐group comparisons, statistical analyses were performed to compare the effects of *Aloe vera* and Doxorubicin between the 2D and 3D cell culture models at each time point and concentration. While these comparisons are not shown in the main figure to preserve visual clarity, significant findings are outlined below. For *Aloe vera*‐treated groups, at the C4 concentration (12.5 mg/mL), 2D cultures exhibited significantly higher cell viability than 3D cultures at both 48 h (*p* < 0.05) and 72 h (*p* < 0.01). This difference is consistent with the peak viability seen in 2D monolayers at this dose, possibly due to enhanced proliferative response in 2D settings with unrestricted nutrient diffusion. However, no significant difference was found at 24 h (C4–2D vs. C4–3D, *p* > 0.05), suggesting that the divergence becomes more pronounced over time. In contrast, Doxorubicin treatment resulted in no statistically significant differences between 2D and 3D models at lower concentrations (C2–C4) across all time points (*p* > 0.05). At higher concentrations (C6–C7), 3D cultures retained slightly higher viability (~10%–15%) than 2D, but the difference was only marginally significant at 72 h (*p* < 0.05) and not at earlier time points. This suggests that while the 3D structure may confer some degree of temporal buffering against chemotoxicity, Doxorubicin effectively suppresses cell viability in both systems.

**Figure 5:**
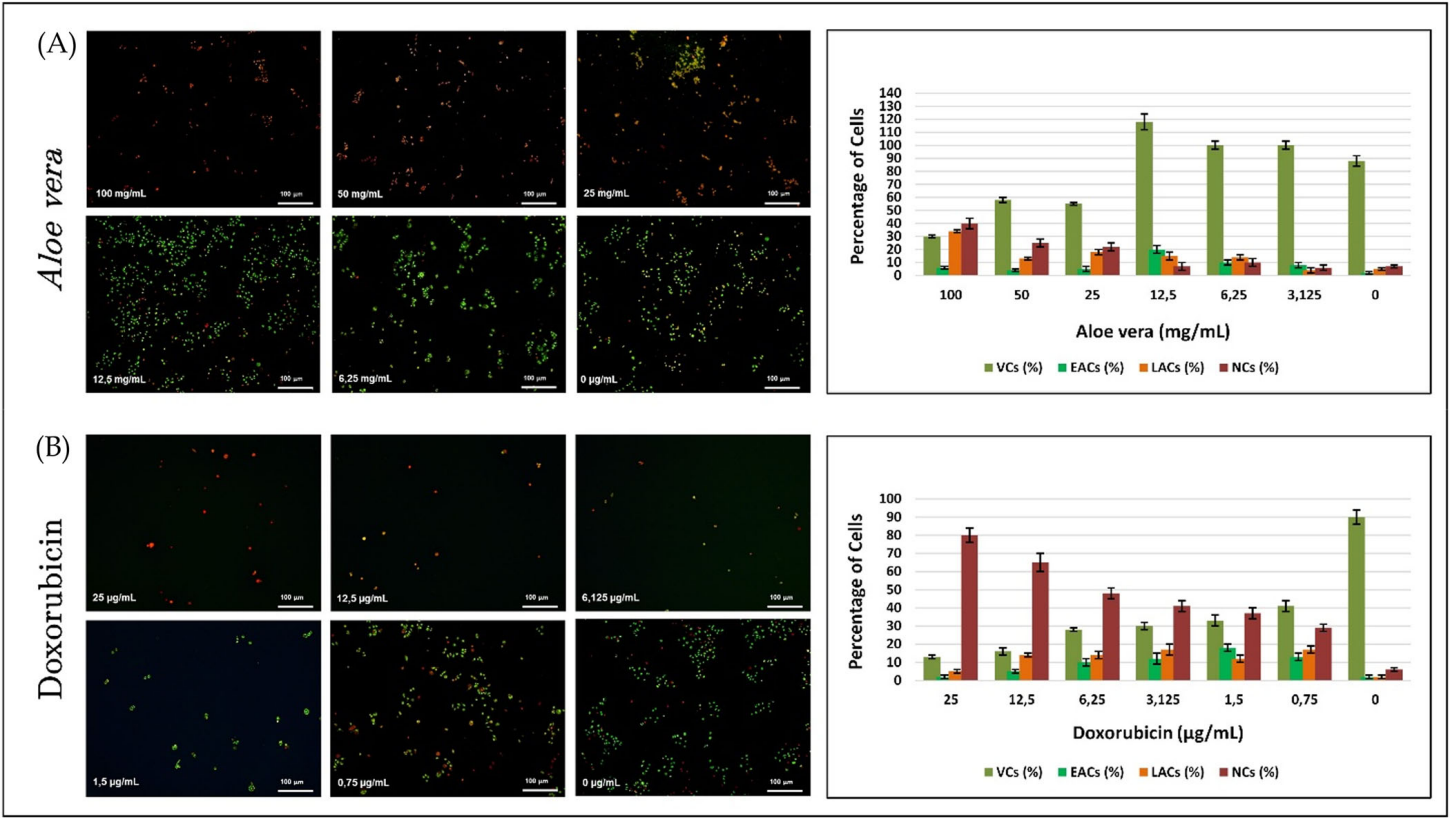
Fluorescence‐based assessment of apoptosis and necrosis in HepG2 cells treated with *Aloe vera* and Doxorubicin for 72 h. (A) Shows fluorescence microscopy images and corresponding quantitative analysis of cells exposed to increasing concentrations of *Aloe vera* (0, 3.125, 6.25, 12.5, 25, 50, and 100 mg/mL). (B) Presents equivalent data for Doxorubicin‐treated cells (0, 0.75, 1.5, 3.125, 6.125, 12.5, and 25 µg/mL). Green fluorescence indicates viable cells (AO‐positive, PI‐negative), early apoptotic cells appear green with nuclear condensation, late apoptotic cells exhibit orange/red fluorescence with nuclear fragmentation, and necrotic cells display intense red staining. The bar graphs reflect the percentages of viable cells (VC), early apoptotic cells (EAC), late apoptotic cells (LAC), and necrotic cells (NC) for each treatment condition. Data are presented as mean ± standard deviation (*n* = 5).

Following the cytotoxicity studies, the apoptotic and necrotic effects of the molecules on HepG2 cells were examined using AO/PI double staining. The tests were conducted on the third day of the culture for the 2D culture studies. The staining images and the percentages of viable cells, early apoptotic cells, late apoptotic cells, and necrotic cells calculated based on the staining results are presented in Figure 5.

*Aloe vera* treatment induced a proliferative response in HepG2 cells at lower concentrations (≤ 12.5 mg/mL), as reflected by a notable increase in the number of green‐stained viable cells. This effect was most pronounced at 12.5 mg/mL, correlating with the highest observed viability in the accompanying bar graph. However, at concentrations exceeding 12.5 mg/mL, a dose‐dependent rise in apoptotic cell populations was detected, with both early and late apoptosis becoming more prominent. At the highest tested dose of 100 mg/mL, late apoptosis and necrosis occurred at comparable levels, and overall cell viability dropped to approximately 30%. In contrast, Doxorubicin exhibited a strong antiproliferative and cytotoxic effect, initiating a rapid decline in viable cell density starting from 0.75 µg/mL. As the dose increased, the proportion of necrotic cells rose markedly, becoming the dominant cell death mechanism at 25 µg/mL, where viability was reduced to around 10%. These findings indicate that while *Aloe vera* exerts a concentration‐dependent proliferative effect at lower doses and induces apoptosis at higher concentrations, Doxorubicin predominantly induces necrosis, particularly at higher doses, and exhibits a more potent cytotoxic effect compared to *Aloe vera*.

**Figure 6:**
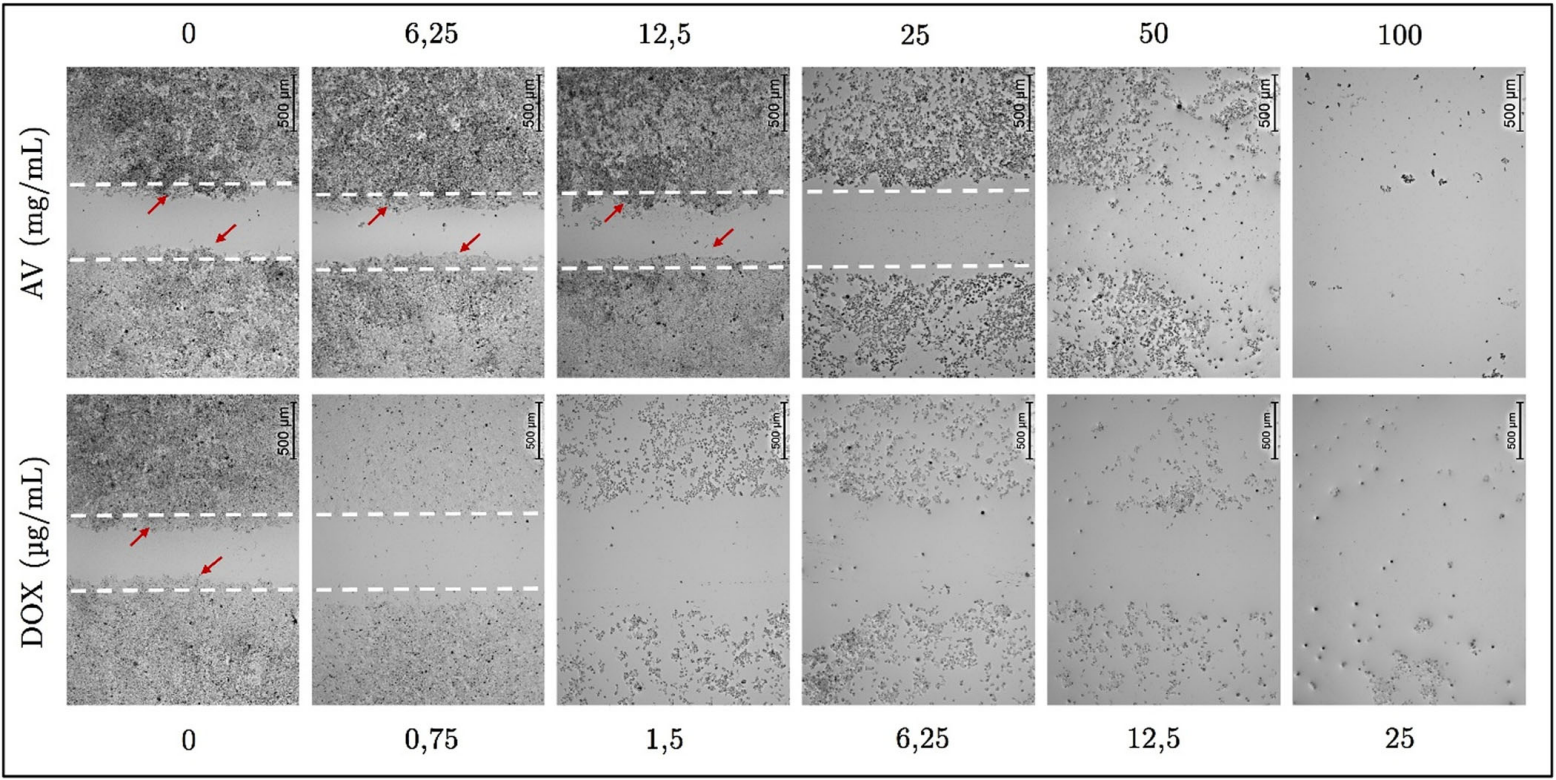
Representative wound healing assay images illustrating the migratory capacity of HepG2 cells after 24 h of treatment with *Aloe vera* or Doxorubicin. The upper row displays Doxorubicin‐treated groups (0, 0.75, 1.5, 3.125, 6.25, 12.5, and 25 µg/mL), while the lower row shows *Aloe vera*‐treated groups (0, 3.125, 6.25, 12.5, 25, 50, and 100 mg/mL). A confluent monolayer was scratched at baseline (0 h), and cell migration into the wound area was assessed at 24 h. The white dashed lines indicate the initial wound boundaries, and red arrows highlight the leading edge of migrating cells.

A wound‐healing assay was conducted to assess the migration capacity of treated and untreated cells, with scratch closure monitored at 24 h and presented in Figure 6.

As shown in Figure 6, *Aloe vera* treatment exhibited a concentration‐dependent effect on cell migration in the wound‐healing assay. At lower dosages (3.125–12.5 mg/mL), significant closure of the scratch region was noted after 24 h, suggesting improved migratory ability and potential proliferative assistance. Nonetheless, at elevated dosages (≥ 25 mg/mL), this impact steadily lessened, resulting in a noticeable reduction in wound closure. These data indicate that *Aloe vera* may promote cell migration at low dosages, while inhibiting motility at higher concentrations. Doxorubicin treatment led to negligible wound healing, even at the lowest dosage examined (0.75 µg/mL), with the scratch gap predominantly staying open under all conditions. This absence of cellular movement corroborates Doxorubicin's strong anti‐migratory and cytotoxic characteristics, consistent with the viability and apoptosis findings. Despite the absence of statistical quantification, the qualitative distinctions noted underscore the dose‐dependent behavioral divergence between *Aloe vera* and Doxorubicin.

**Figure 7:**
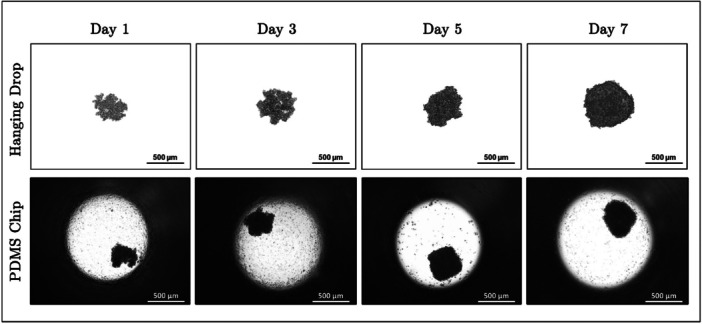
Light microscopy images of spheroids were formed using the conventional hanging drop technique, and spheroids were generated within polydimethylsiloxane (PDMS) microfluidic chips on different culture days.

In the later stages of the study, the comparative effects of *Aloe vera* and Doxorubicin were examined in 3D cultures by using microfluidic chips. For this purpose, spheroids were successfully generated within the chip, and to evaluate the efficiency of spheroid formation, they were compared with spheroids produced using the conventional hanging drop technique with the same cell density. Light microscopy images were taken under identical conditions over a 7‐day culture period to allow for a direct comparison of spheroid morphology and structural integrity between the two methods, and the corresponding images are presented in Figure 7.

The comparison between spheres formed by the conventional hanging drop technique and those formed in PDMS microfluidic chips reveals important observations regarding sphere formation over time. In the conventional hanging drop technique, spheroids gradually become more compact as the culture progresses. They maintain a uniform and dense 3D structure, confirming the reliability of this method for forming spheroids in a static environment. Similarly, spheroids formed within the PDMS microfluidic chip also exhibit a well‐organized, compact structure, highlighting the ability of the chip to support 3D tissue‐like architecture. While the spheroids appear smaller at later time points in the chip group, this is attributed not to structural degradation but to enhanced compaction and cellular organization facilitated by flow‐mediated removal of loosely attached or apoptotic cells. This interpretation is supported by our COMSOL flow simulation analysis, which confirmed that the shear stress within the chip remains below the thresholds known to cause physical disruption of spheroids. Previous studies have also demonstrated that controlled perfusion in microfluidic systems can enhance spheroid quality by promoting the clearance of cellular debris and improving mechanical cohesiveness (Huang et al. 2020; Nashimoto et al. 2017; Ong et al. 2017). Moreover, the presence of dead cells and debris around the spheres in the wells is an important observation, indicating natural cell turnover and dynamic interactions with the surrounding microenvironment. Therefore, the PDMS chip not only facilitates spheroid formation, but also contributes to maintaining spheroid stability by enabling low‐shear, flow‐assisted clearance of undesirable cellular material.

**Figure 8:**
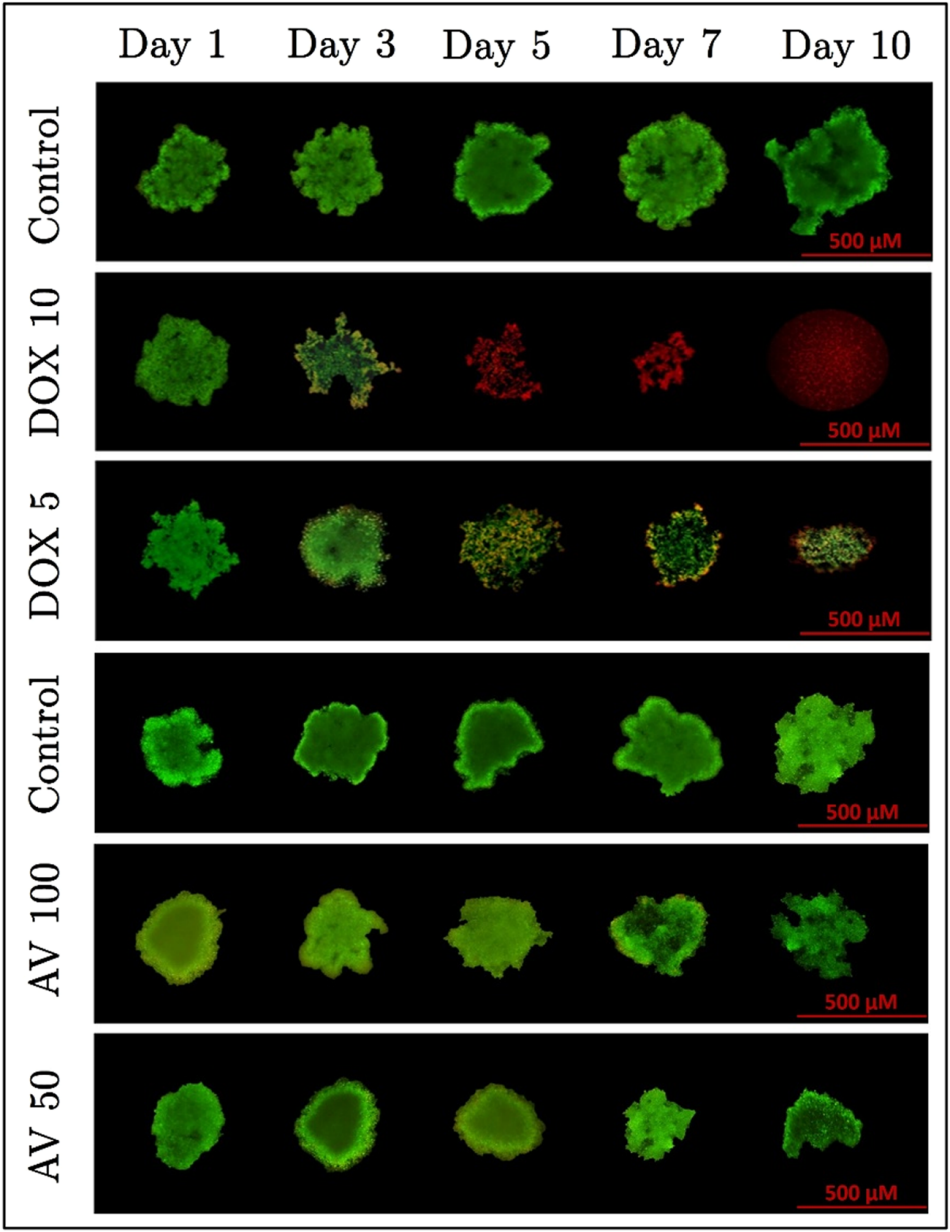
Apoptosis and necrosis assessment of three‐dimensional (3D) spheroids treated with *Aloe vera* (50 mg/mL and 100 mg/mL) and Doxorubicin (5 µg/mL and 10 µg/mL) inside a polydimethylsiloxane (PDMS)‐based microfluidic chip. Spheroids were stained using acridine orange (AO)/propidium iodide (PI) double staining to evaluate cell viability, apoptosis, and necrosis. Green fluorescence represents viable cells, orange indicates early/late apoptotic cells, and red denotes necrotic cells. In the *Aloe vera* 100 mg/mL group, which represents a supra‐IC50 concentration, a gradual decrease in spheroid size and increased apoptosis and necrosis were observed qualitatively. In the Doxorubicin 10 µg/mL group, also a supra‐IC50 dose, necrosis became predominant, with almost no cells remaining by Day 5. The results demonstrate the dose‐dependent effects of both agents in a 3D culture system, with distinct apoptotic and necrotic pathways triggered at supra‐IC50 concentrations. Red scale bars indicate 500 µm and serve as a visual reference for spheroid size across all groups and time points.

To better understand the cytotoxic effects of *Aloe vera* and Doxorubicin in a more physiologically relevant 3D environment, an apoptosis and necrosis assay was conducted on spheroids generated within the microfluidic chip, and the staining results are presented in Figure 8. This assay aimed to evaluate the extent of cell death, apoptosis, and necrosis induced by different concentrations of these agents, providing insights into their effectiveness in a 3D culture system that closely mimics in vivo tumor behavior. The rationale for selecting the doses of *Aloe vera* (100 mg/mL and 50 mg/mL) and Doxorubicin (10 µg/mL and 5 µg/mL) is based on the IC50 values previously obtained in our study under 3D culture conditions. For *Aloe vera*, the IC50 value at 72 h in 3D culture was determined to be 31.25 mg/mL. Based on this, both selected doses (50 mg/mL and 100 mg/mL) are above the IC50 threshold. However, these doses were intentionally chosen to reflect the transition from moderate apoptotic activity to more pronounced necrotic responses, as observed in our viability and fluorescence microscopy data. Given the complex, plant‐derived nature of *Aloe vera* and its biphasic dose–response behavior (hormesis), supra‐IC50 concentrations were found to be more appropriate to evaluate dose‐dependent cell death mechanisms to visualize apoptotic‐necrotic changes in spheroids. Thereby, the 50 mg/mL dose represents a moderately elevated level relative to the IC50, allowing observation of early apoptotic effects, while 100 mg/mL provides a clear necrotic signature. For Doxorubicin, the IC50 value in 3D culture at 72 h was found to be 8.33 µg/mL. Thus, the 5 µg/mL dose was chosen to evaluate effects below the IC50, where a lower level of cytotoxicity is expected, while the 10 µg/mL dose was selected to explore the effects above the IC50, where significant cell death through apoptosis and necrosis is anticipated. These doses were strategically chosen to comprehensively analyze both agents' effects on spheroid viability, apoptosis, and necrosis within the 3D culture system, ensuring a thorough assessment of cytotoxic mechanisms, even though the *Aloe vera* doses fall within a supra‐IC50 range.

In the *Aloe vera* (100 mg/mL) group, the size of the spheroids gradually decreased over time, and both apoptosis and necrosis were significantly increased. Apoptosis, indicated by the orange staining, was particularly dominant on Days 5 and 7, suggesting that cell death was primarily triggered via apoptotic pathways. However, due to the high dose, necrosis was also evident. Cell viability was better preserved in the 50 mg/mL *Aloe vera* group, and the spheroids largely maintained their size. Apoptosis rates were lower in this group, though still close to the 72‐h IC50 value of 31.25 mg/mL, reflecting moderate cytotoxic effects. In the Doxorubicin (10 µg/mL) group, almost no cells remained in the environment from Day 5 onward. Necrosis, indicated by the red staining, was highly predominant, with most cells becoming necrotic. In contrast, the 5 µg/mL Doxorubicin group preserved cell viability to a greater extent, with lower levels of apoptosis and necrosis observed. Given the IC50 value of 8.33 µg/mL, these results indicate that cytotoxic effects were more limited at lower doses. Overall, the obtained results demonstrate that *Aloe vera* predominantly induces apoptosis, while Doxorubicin triggers more necrosis. The shrinking of spheroids and the appearance of necrosis in the *Aloe vera* 100 mg/mL group reflect the effects of high concentrations, while the rapid and extensive cell death observed from Day 5 onward in the Doxorubicin 10 µg/mL group highlights the potent and swift cytotoxic impact of this dose. These findings align with the IC50 values obtained in 3D culture, confirming the dose‐dependent effects of both agents.

**Figure 9:**
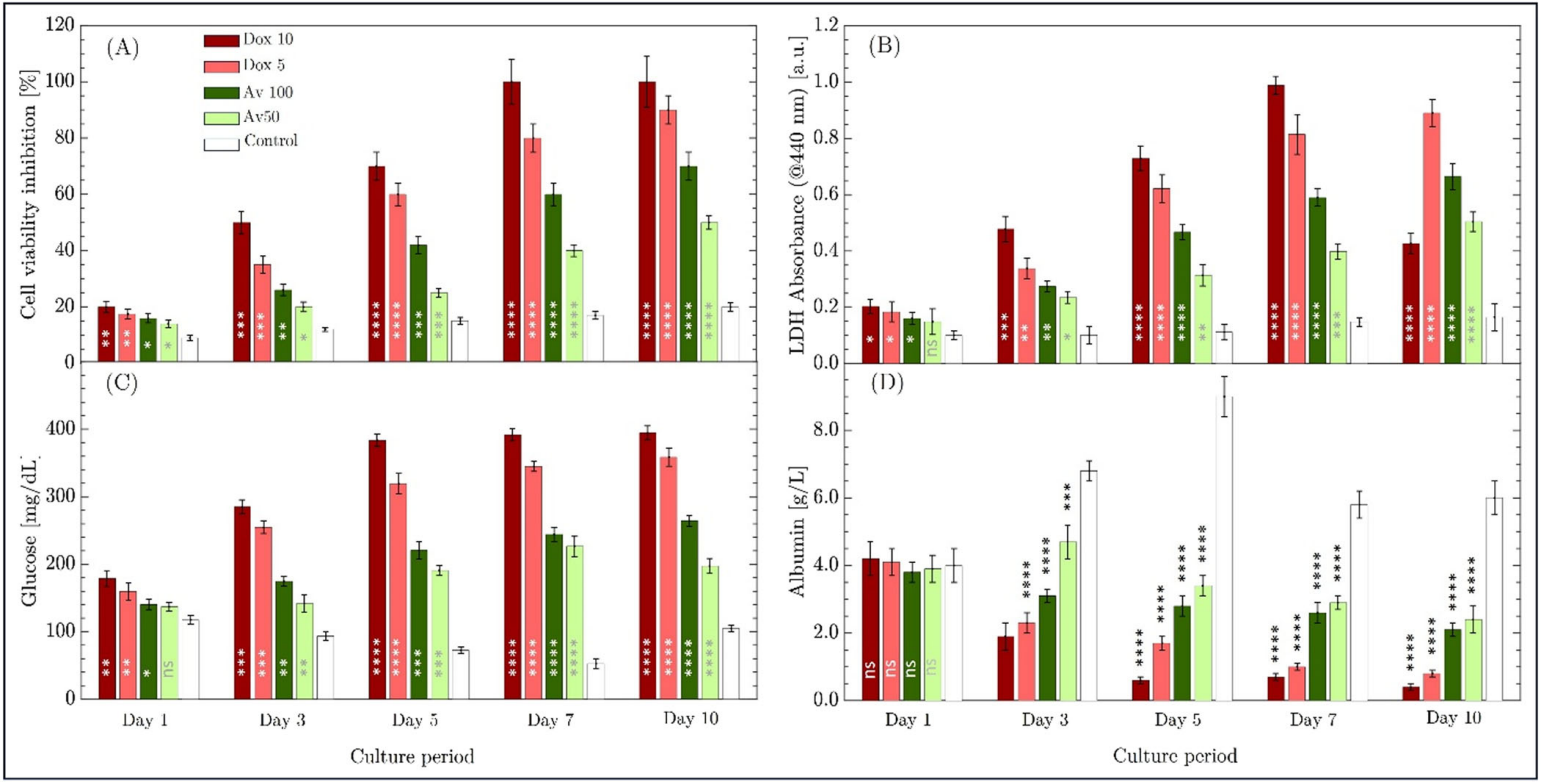
Evaluation of cellular responses in 3D spheroids treated with different concentrations of Doxorubicin (Dox 10, Dox 5) and *Aloe vera* (AV 100, AV 50) over a 10‐day culture period. (A) Cell viability inhibition measured via Alamar Blue assay, showing significant reductions in viability across treatment groups compared to control, with Doxorubicin causing a more rapid and pronounced inhibition. (B) LDH release into the culture medium as an indicator of membrane damage, with Doxorubicin causing a significant increase in LDH levels, indicative of necrosis, while *Aloe vera* induced a more gradual release, consistent with apoptotic cell death. (C) Remaining glucose levels in the culture medium, highlighting reduced glucose consumption in the Doxorubicin‐treated spheroids due to cell death, while *Aloe vera‐*treated spheroids showed a delayed but progressive reduction in glucose consumption. (D) Albumin secretion as a measure of liver function, with Doxorubicin causing a rapid decline in albumin production, and *Aloe vera* exerting a more gradual inhibitory effect on metabolic activity. Data are presented as mean ± standard deviation (*n* = 3). Statistical significance shown was evaluated using one‐way ANOVA followed by Tukey's post hoc test. Significant differences compared to the control group at each time point are indicated as follows: *p < 0.05, **p < 0.01, ***p < 0.001, **p < 0.0001, and ns = not significant. ANOVA, analysis of variance; LDH, lactate dehydrogenase.

The inhibitory effect of *Aloe vera* and Doxorubicin on the proliferation of cells in spheroids was assessed during the culture period using Alamar Blue analysis, with results presented in Figure 9A. Repeated‐measures ANOVA (two‐way) revealed that both time and treatment significantly affected cell viability inhibition (*p* < 0.0001), with a noteworthy interaction between the two variables (*p* = 0.0021). Tukey's post hoc comparisons indicated significant differences between early time points (Day 1) and subsequent stages (Day 5, Day 7, and Day 10) among all treatment groups. Doxorubicin‐treated cells (Dox 10 and Dox 5) exhibited swift and dramatic increases in inhibition, with significant differences observed from Day 1 to Day 5 and Day 10 (*p* < 0.0001). *Aloe vera* treatments demonstrated a gradual but continuous inhibitory effect, with significant differences noted from Day 1 to Day 7 and Day 10 (*p* < 0.0001). The control groups exhibited a gradual increase in inhibition over time, albeit to a lesser extent. Significantly, for Doxorubicin, inhibition reached equilibrium after Day 7, as no substantial variations were detected between Day 7 and Day 10 (*p* > 0.05). The data indicate that Doxorubicin elicits a robust, immediate cytotoxic reaction, whereas *Aloe vera* demonstrates a more delayed effect, with potency augmenting with time. These findings highlight the fluctuating effectiveness of treatment over time, with implications for improving therapeutic approaches. At the conclusion of the 10‐day culture period, the control group exhibited approximately 20% viability inhibition, which was ascribed to cell mortality caused by inherent challenges and diffusion constraints in mass transfer from the proliferation zone on the spheroid's outer wall to the innermost necrotic zone during spheroid growth (Bull et al. 2020; Khan et al. 2022).

Figure 9B depicts the results of LDH measurements in the supernatants collected from spheroids exposed to different drug treatments. LDH is a stable cytoplasmic enzyme present in all cells and is rapidly released into the culture medium when the plasma membrane is compromised, making it a reliable marker of cytotoxicity and cellular damage, including apoptosis and necrosis (Castiglione et al. 2024). Based on the repeated‐measures ANOVA (two‐way) statistical findings, both time and treatment groups had a significant effect on LDH activity (*p* < 0.0001), with a significant interaction between these two factors, indicating that the effect of treatments on LDH release varied over time. Tukey's post hoc comparisons revealed that LDH release significantly increased between early time points (Day 1) and later stages (Day 5, Day 7, and Day 10) (*p* < 0.001), suggesting cumulative cellular damage over the course of the experiment. However, no significant difference was observed between Day 7 and Day 10 (*p* > 0.05), indicating that LDH release stabilized in the later stages. In terms of treatment effects, Doxorubicin induced a more immediate and substantial increase in LDH activity, reflecting a higher level of necrosis and rapid cell death. In contrast, *Aloe vera* treatment resulted in a more gradual increase in LDH release, consistent with the induction of apoptosis rather than necrosis. Fluorescence microscopy findings also corroborate this observation, showing a lower degree of necrosis in *Aloe vera*‐treated spheroids, where apoptosis or early apoptosis predominates. The lower LDH levels in the *Aloe vera*‐treated groups suggest that the cells undergo primarily apoptotic pathways, which do not involve the breakdown of the cell membrane to the same extent as necrosis. As a result, less LDH is released into the environment compared to Doxorubicin‐treated groups, where necrotic cell death leads to a more pronounced release of LDH into the culture medium. Overall, these findings highlight the different modes of cell death induced by Doxorubicin and *Aloe vera*, with Doxorubicin leading to rapid necrosis and *Aloe vera* causing a slower, apoptosis‐driven process.

Figure 9C depicts the results of glucose measurements conducted on the same supernatants obtained from spheroids subjected to various drugs at differing concentrations for LDH analysis. The repeated‐measures ANOVA (two‐way) statistical analysis revealed that both time and treatment groups had a substantial impact on the residual glucose levels in the culture medium (*p* < 0.0001). Tukey's post‐hoc comparisons indicated that in the control group, residual glucose levels dramatically diminished from Day 1 to later time points (Day 3, Day 5, and Day 7, all *p* < 0.05), suggesting active glucose use by live cells. Extremely low glucose concentrations observed in the continually replenished culture medium suggested that the untreated spheroids preserved their viability and consistently metabolized glucose during the culture period. No significant variation was noted between Day 1 and Day 10 (*p* = 0.6078), indicating stabilization of glucose levels, perhaps attributable to the cell death observed in the later phases of the culture. In the Doxorubicin‐treated groups, specifically Dox 10 and Dox 5, the residual glucose levels dramatically elevated over time, with considerable differences observed between Day 1 and all subsequent time points (*p* < 0.05), indicating decreased glucose utilization attributable to cellular apoptosis. *Aloe vera* treatments (both AV 100 and AV 50) exhibited a progressive rise in residual glucose levels, with significant differences noted between Day 1 and Days 7 and 10 in the AV 100 group (*p* < 0.05), suggesting a delayed although consistent suppression of glucose utilization. These data indicate that Doxorubicin swiftly reduces glucose intake by promoting cell death, whereas *Aloe vera* has a more gradual inhibitory impact on metabolic activity over time.

Figure 9D illustrates the results of albumin quantifications conducted on the supernatants obtained from spheroids subjected to various doses of drugs during the culture period. Albumin is an essential protein that maintains pressure from oncotic cells and facilitates molecular transport in the blood; its release is frequently utilized as an indicator of liver function and cellular viability in three‐dimensional culture models. The repeated‐measures ANOVA (two‐way) statistical analysis revealed that both time and treatment groups had a substantial impact on albumin secretion (*p* < 0.0001). Tukey's post hoc comparisons indicated that in the control group, significant reductions in albumin secretion occurred between Day 1 and later time periods, especially between Day 1 and Day 5 (*p* < 0.0001). This decrease indicates that as the culture advances, metabolic activity inherently decreases. In the Doxorubicin‐treated groups (Dox 10 and Dox 5), albumin secretion decreased significantly over time, with notable changes observed between Day 1 and subsequent time points (*p* < 0.05). This decrease is likely attributable to the cytotoxic effects of Doxorubicin, resulting in cell death and a significant drop in metabolic activity. Conversely, the *Aloe vera*‐treated groups had a more gradual decline in albumin secretion, with notable reductions occurring mostly between Day 1 and Day 7, as well as Day 10 (*p* < 0.05), indicating a slower suppression of cellular activity. The reduction in albumin secretion in 3D spheroids subjected to anticancer medicines highlights the decline in cell viability and function, as these spheroids undergo considerable stress and apoptosis when exposed to such agents. These data indicate that Doxorubicin has a swift and strong effect on cellular activity, whereas *Aloe vera* exhibits a more gradual and restrained impact on albumin formation and cellular metabolism over time.

The results of the cell viability inhibition, LDH activity, glucose consumption, and albumin secretion assays collectively demonstrate the varying cytotoxic effects of Doxorubicin and *Aloe vera* on 3D spheroids over time. Doxorubicin, especially at higher doses, had a swift and considerable effect across all assays, characterized by prompt and substantial suppression of cell viability, increased LDH release, and reduced glucose consumption and albumin secretion. The findings suggest necrosis‐induced cell death, as seen by increased LDH levels and a significant reduction in consumption of glucose and albumin secretion, indicating metabolic cessation and compromised cellular integrity. *Aloe vera* treatments demonstrated a more progressive impact on spheroid metabolism, exhibiting delayed although consistent suppression of cell survival and metabolic function. Compared to Doxorubicin, LDH release increased more slowly, indicating apoptosis rather than necrosis, which the fluorescence microscopy data supported. The progressive decrease in glucose utilization and albumin release confirms the idea that *Aloe vera* promotes a more gradual, apoptosis‐mediated cell death process, maintaining metabolic function for an extended period compared with Doxorubicin.

An important consideration in interpreting these results is the effect of drug penetration limitations in 3D spheroid cultures (LaBonia et al. 2016). Due to the dense structure of spheroids, drug diffusion may be restricted, especially in the deeper layers (Biju et al. 2023). This diffusion limitation likely contributed to the more immediate cytotoxic effects observed with Doxorubicin in the outer spheroid layers, while the inner layers exhibited delayed cell death. This explains the plateau in LDH release and glucose consumption in the later phases of the culture period. In the same vein, the gradual effects of *Aloe vera* may have been influenced by these penetration challenges, as the drug may have required a longer time to reach the center of the spheroids, resulting in a slower accumulation of apoptotic markers. These results underscore the significance of taking drug penetration limitations into account when evaluating the efficacy of anticancer agents in 3D culture models, as they can substantially affect the timing and magnitude of observed cytotoxic effects. Doxorubicin, particularly at the higher dose, exhibited a rapid and significant impact in all assays, resulting in early and substantial inhibition of cell viability, increased LDH release, and reduced glucose consumption and albumin secretion. These results suggest that necrosis‐driven cell death is the cause, as evidenced by the significant decrease in glucose consumption and albumin secretion and the elevated LDH levels. This indicates both metabolic closure and a loss of cellular integrity. Conversely, the spheroid metabolism was more gradually affected by *Aloe vera* treatments, which demonstrated a progressive but consistent reduction in cell viability and metabolic function. The fluorescence microscopy results corroborated the notion that apoptosis was more prevalent than necrosis, as LDH release increased at a slower pace than Doxorubicin. The notion that *Aloe vera* induces a slower, apoptosis‐driven mechanism of cell death, which maintains metabolic function for a longer duration than Doxorubicin, is further illustrated by the progressive decrease in glucose consumption and albumin secretion.

The effectiveness of drug penetration in 3D spheroids is influenced by several factors, including molecule size, cellular uptake mechanisms, and the structural properties of the spheroids themselves (Sokolova et al. 2019). Doxorubicin, with a molecular weight of approximately 543.52 g/mol, is able to penetrate cells relatively quickly due to its ability to diffuse passively through the cell membrane and its involvement in active transport mechanisms (Kciuk et al. 2023). This results in cytotoxic effects that are both rapid and severe, particularly in the outer layers of the spheroids. Conversely, the larger bioactive components of *Aloe vera*, which include polysaccharides and phenolic compounds, are absorbed by cells at a slower rate through mechanisms such as receptor‐mediated endocytosis. This leads to a cytotoxic response that is delayed, which is in accordance with the apoptosis‐driven effects that were observed in this study.

Furthermore, the size and density of 3D spheroids, which are inherent structural characteristics, further influence drug penetration (Singh et al. 2020). Drug concentrations may be substantially reduced in the inner necrotic core of larger spheroids, which presents a greater barrier to effective diffusion. Drug stability is also a critical factor, as less stable compounds may degrade before reaching the innermost cells, thereby diminishing their efficacy (Ashutosh Kumar Yadav et al. 2023). Drug efficacy can be further compromised by microenvironmental factors, including hypoxia and acidic pH, which disrupt cellular metabolism and diminish the drug's stability over time (Bakshi et al. 2024). Collectively, these factors underscore the significance of taking into account both molecular characteristics and spheroid architecture when assessing the therapeutic potential of anticancer agents, as they have a direct impact on drug distribution, cellular absorption, and overall effectiveness in 3D models.

Building on the results of the present study, future studies should investigate a range of avenues to further our comprehension of the therapeutic benefits of *Aloe vera* and Doxorubicin in 3D spheroid models. A critical focus is the examination of the molecular mechanisms that govern the apoptotic and necrotic cell death pathways triggered by these agents. Identifying the specific signaling pathways, including caspase activation and DNA damage responses, will enhance understanding of the varying cytotoxic effects observed. Moreover, prioritizing the optimization of drug delivery strategies to address the challenges of drug penetration in dense 3D spheroids is essential. This involves investigating advanced drug carriers, such as nanoparticle‐based systems, to improve drug distribution and efficacy. Microenvironmental factors, including hypoxia, pH gradients, and nutrient availability within spheroids, require further investigation due to their potential impact on drug efficacy and cell death pathways. Transitioning these findings into in vivo studies is essential for evaluating the interactions of these treatments with the tumor microenvironment and for assessing their clinical potential.

## 4 Conclusion

This study points out the significant possibility of *Aloe vera* as a natural anticancer agent, especially when compared to the established chemotherapeutic agent, Doxorubicin. Utilizing a PDMS‐based microfluidic platform, we demonstrated the formation of compact, viable 3D HepG2 spheroids, offering a physiologically relevant model for drug testing. The comparison of *Aloe vera* and Doxorubicin in 3D spheroid cultures indicates differing effects, with *Aloe vera* exhibiting concentration‐dependent cytotoxicity and Doxorubicin displaying a more pronounced antiproliferative effect. This study confirms the efficacy of *Aloe vera* within a 3D culture system and illustrates the application of PDMS‐based microfluidic devices in cancer research. These platforms provide a cost‐effective, reproducible, and scalable approach for drug screening and personalized medicine applications, especially regarding liver cancer. This study offers insights into the potential of natural compounds, such as *Aloe vera*, for future anticancer therapies and emphasizes the benefits of employing microfluidic systems for enhanced accuracy and reliability in drug testing within three‐dimensional tumor models.

## Ethics Statement

The authors have nothing to report.

## Conflicts of Interest

The authors declare no conflicts of interest.

## Data Availability

The data set used and analyzed during the current study are available from the corresponding author on reasonable request.
